# Acute cholecystitis during pregnancy: a systematic review and meta-analysis

**DOI:** 10.1186/s12893-026-04087-5

**Published:** 2026-08-07

**Authors:** Georgios K. Kyrtsonis, Stergios Bobotis, Georgios Geropoulos, Konstantinos Katsikas Triantafyllidis, Imran Tariq Hassan, Evgenia-Stavroula Vougiouklaki, Apostolia Galani, Konstantinos S. Kechagias

**Affiliations:** 1 Department of General Surgery, Croydon University Hospital, Croydon, London, UK; 2 Department of Obstetrics and Gynaecology, Tunbridge Well and Maidstone NHS Trust, Tunbridge Wells, UK; 3 Department of General Surgery, Watford General Hospital, Watford, UK; 4 Department of Nutrition and Dietetics, Homerton University Hospital Foundation Trust, London, UK; 5 Brighton and Sussex Medical School, Brighton, UK; 6 School of Medicine, National and Kapodistrian University of Athens, Athens, Greece; 7 Department of Metabolism, Digestion and Reproduction, Faculty of Medicine, Imperial College London, London, UK; 8 Department of Obstetrics and Gynaecology, The Hillingdon Hospitals NHS Foundation Trust, Uxbridge, UK

**Keywords:** Pregnancy, Gestation, Gallbladder, Acute abdomen, Surgery

## Abstract

**Background:**

Acute cholecystitis is an uncommon non-obstetric surgical emergency in pregnancy, yet guidance on management remains inconsistent. This systematic review and meta-analysis examined presentation, diagnostics, management, and outcomes of pregnant patients diagnosed with acute cholecystitis.

**Methods:**

PubMed, Web of Science, and Scopus were searched from inception to February 2024 (PROSPERO CRD42022350290). Eligible studies reported acute cholecystitis in pregnancy with individual patient data or extractable cohort data. Risk of bias was assessed in duplicates. Descriptive statistics summarised individual data, and random-effects meta-analysis generated pooled risk ratios (RR) with 95% confidence interval (CI).

**Results:**

Ninety studies (1964–2023) were included: 48 case reports/series (*n* = 118), 19 descriptive cohorts (*n* = 5,702), 19 cohorts comparing operative versus non-operative care (*n* = 91,955), and 4 cohorts comparing laparoscopic versus open surgery (*n* = 62,620). Among cases with symptom data (*n* = 75), right-upper-quadrant pain (40%), vomiting (33%), nausea (29%), and fever (17%) predominated. Imaging (*n* = 92) most often showed gallstones (42%) and ultrasonographic cholecystitis or wall thickening (21%). Of the cases with management data (*n* = 93), surgery was performed in 81 (laparoscopic 71/81; percutaneous drainage 10/81; open 1/81);12 were managed non-operatively. Of 83 with outcomes, 84% had uncomplicated courses. Meta-analysis found no significant differences between operative and non-operative care for caesarean delivery, preterm birth, postpartum haemorrhage, or composite complications, but venous thromboembolism risk was lower with surgery (RR 0.55, 95% CI 0.37–0.82), although this outcome was reported by only two studies.

**Conclusions:**

Ultrasound-based diagnosis and laparoscopic cholecystectomy appear safe across all trimesters of pregnancy, with obstetric outcomes comparable to non-operative management. Operative management was associated with a lower observed risk of venous thromboembolism, although this finding was based on limited evidence. Further prospective studies are needed to optimise patient selection and surgical timing.

**Supplementary Information:**

The online version contains supplementary material available at https://doi.org/10.1186/s12893-026-04087-5.

## Background

Surgical emergencies during pregnancy are rare with a reported incidence of around 2 per 1,000 pregnancies [1–3]. Gallbladder disease constitutes the second most common surgical emergency in pregnant females, after acute appendicitis, with its incidence ranging from 5 to 8 per 10,000 [4]. Pregnant women are at increased risk of developing biliary sludge and gallstone formation in the gallbladder, due to weight gain and hormonal changes during pregnancy [5]. Thus, nearly 8% of pregnant women form new gallstones by the third trimester, but only 1% develop symptoms. Of those, less than 10% develop complications including acute cholecystitis, choledocholithiasis, and acute pancreatitis [6].

Gallstone disease may present across a spectrum of severity, ranging from biliary colic to acute cholecystitis. Biliary colic typically manifests as episodic right upper quadrant or epigastric pain, often accompanied by nausea, vomiting, bloating, and food intolerance, with pain that may radiate to the back or right shoulder [7, 8]. Acute cholecystitis, most commonly caused by gallstone obstruction of the cystic duct, presents with similar but generally more severe and persistent symptoms, frequently associated with systemic inflammatory features such as fever [7, 8]. Right upper quadrant tenderness and a positive Murphy’s sign are characteristic clinical findings and may aid diagnosis in the appropriate clinical context [7, 8].

Diagnosis of acute cholecystitis relies on a combination of clinical assessment and imaging findings [8–10]. Abdominal ultrasound is the preferred first-line imaging modality because of its diagnostic accuracy, widespread availability, and safety during pregnancy [8–10]. Management strategies vary according to gestational age, symptom severity, and disease progression, and may include supportive treatment, antibiotics, endoscopic retrograde cholangiopancreatography (ERCP), percutaneous gallbladder drainage, laparoscopic cholecystectomy, or open cholecystectomy [8–10]. Despite advances in surgical and perioperative care, the optimal management of acute cholecystitis during pregnancy remains debated, particularly with regard to the timing of intervention across different trimesters [9, 10]. Furthermore, evidence specifically addressing acute cholecystitis during pregnancy remains limited, highlighting the need for a comprehensive synthesis of the available literature.

## Methods

This review was reported based on the “Preferred Reporting Items for Systematic Reviews and Meta-Analyses” (PRISMA) guidelines and the protocol was registered in PROSPERO online database (CRD: 42022350290).

### Literature search

Two reviewers (GK, KKT) independently conducted a comprehensive search of PubMed, Web of Science, and Scopus from database inception through February 2024. The search included the following terms: (cholecystitis OR gallbladder inflammation OR gallbladder empyema) AND (pregnanc* OR gestation). No limits were applied with respect to language, geographical setting, or study design. Backward citation searching (snowballing) was also performed through manual screening of the reference lists of eligible studies and relevant reviews. Discrepancies in the literature search process were resolved by a third investigator (GG).

### Eligibility criteria

Studies reporting cases of acute cholecystitis occurring during pregnancy were eligible for inclusion. We considered all primary study designs (including case reports, case series, and observational cohorts) provided that relevant clinical data were available. Case reports and case series were included to capture detailed clinical information regarding presentation, diagnosis, management, and rare maternal or fetal outcomes, but were analysed separately from comparative cohort studies. Reviews, conference abstracts without full manuscripts, and non-peer-reviewed publications were excluded.

### Data extraction and handling

Data were extracted independently by two reviewers (GK, KKT) using a predefined data collection form. Extracted variables included patient demographics, gestational age, clinical presentation, diagnostic findings, treatment approach, and outcomes. Any discrepancies between reviewers were resolved through discussion with a third investigator (GG). The predefined outcomes of interest included clinical presentation, imaging findings, trimester at diagnosis, treatment approach (operative versus non-operative management), and maternal and neonatal outcomes. Comparative maternal outcomes included caesarean delivery, preterm birth, abortion, postpartum haemorrhage, venous thromboembolism, and other reported maternal complications. Outcomes were extracted as reported by the original studies. As standardised outcome definitions were not consistently provided across the included literature, comparative outcomes were analysed according to the definitions used by the original study authors.

### Quality assessment

Risk of bias (RoB) was assessed by two authors independently (KSK and KKT). The critical appraisal checklist provided by the Joanna Briggs Institute (JBI) was employed to evaluate the overall quality of case reports and case series. The assessment was based on the reporting of 8 different elements namely: patient demographics, medical history, health status, physical examination and diagnosis, concomitant therapies, post-intervention health status, drug administration reaction interface. The studies were scored either based on “Yes”, “No”, “Unclear or Not/Applicable” depending on the availability of information for every element. The Methodological Index for Non-Randomized Studies (MINORS) tool was used to assess the quality of cohort studies.

### Data synthesis

Given the descriptive nature of this review, we used descriptive statistics to report demographics and clinical characteristics, with means and standard deviation for continuous variables and frequencies and percentages for binary variables. Regarding the comparative data, effect sizes were pooled as risk ratios (RR) with 95% confidence intervals (95% CI) using a random-effects meta-analysis based on the inverse variance method. Between-study variance was estimated with the DerSimonian and Laird method. Statistical heterogeneity was assessed using the χ² test (*P* < 0.10 considered significant), the I² statistic, and τ². I² values of 30–49% were defined as indicating moderate heterogeneity, and values ≥ 50% as high heterogeneity.

## Results

The initial screening of three electronic databases yielded 665 articles. After the exclusion of duplicates and not relevant studies 134 articles were screened for eligibility. A total of 90 studies were included in this systematic review, published between 1964 and 2023. Of these, 48 were case reports or series providing individual patient data (*n* = 118), 19 were descriptive cohort studies (*n* = 5,702), 19 were comparative studies evaluating operative versus non-operative management (*n* = 91,955), and 4 compared laparoscopic versus open surgical approaches (*n* = 62,620). Geographically, 37 studies were conducted in North America, 25 in Asia, 20 in Europe, 5 in South America, 2 in Oceania, and 1 in Africa, reflecting a broad global distribution of data across diverse healthcare settings and populations (Supplementary material).

### Case reports and series

Among 75 patients with available data, right upper quadrant (RUQ) pain was the most common symptom, reported in 40% (30/75), followed by vomiting in 33% (25/75), nausea in 29% (22/75), and fever in 17% (13/75). Less frequently reported were epigastric pain (9%, 7/75), chills or musculoskeletal symptoms (4%, 3/75), and back pain (1%, 1/75).

Among 92 patients with available imaging data, gallstones were the most common finding, reported in 42% (39/92), including 9% (8/92) with multiple gallstones explicitly mentioned. Imaging suggestive of cholecystitis or a thickened gallbladder wall was present in 21% of patients (19/92), while sludge was observed in 4% (4/92). Involvement of the common bile duct (CBD) was reported in 7% (6/92), and acalculous cholecystitis was noted in 2% (2/92). A positive Murphy’s sign was documented in 3% (3/92). In 22% of cases (20/92), imaging was either unremarkable or not reported.

Of the 93 cases with individual patient data, surgical management was the predominant approach, reported in 81 cases. Among surgically managed patients, the index operation was laparoscopic cholecystectomy in 71 patients, percutaneous cholecystostomy as a bridge in 10, and open cholecystectomy in 1. Additional procedures performed in some of these patients included bile duct exploration (*n* = 2) and exploratory laparotomy (*n* = 2); because several patients underwent more than one procedure, procedure counts sum to more than 81. Duration of admission was explicitly recorded in only two cases, with a mean hospital stay of 7.5 days.

Out of 83 cases with available outcome data, 70 (84%) were reported as having normal or uncomplicated clinical courses, using descriptors such as “no complications,” “uneventful recovery,” or “normal delivery.” The majority of births were vaginal deliveries (*n* = 12), with caesarean section performed in 2 cases. Term delivery occurred in 7 pregnancies, while preterm delivery was reported in 2, including one at 25 weeks following failed tocolysis. Notably, only 2 patients (2.4%) experienced significant complications—one maternal (postoperative small bowel obstruction requiring multiple resections) and one foetal (neonatal death and retinopathy in premature twins). Of the 87 reported pregnancies, caesarean section was performed in 9 cases (1%), and preterm delivery occurred in 4 cases (5%), including one as early as 25 weeks gestation. The remaining pregnancies resulted in uncomplicated vaginal births or were still ongoing at the time of reporting.

### Descriptive cohort studies

A total of 16 studies reported mean maternal age, with values ranging from 22 to 39.2 years. The pooled mean age was 28.5 (± 4.6) years. Gestational age at diagnosis was most commonly in the second trimester. Specifically, among studies reporting trimester of diagnosis, 43.5% (966/2,222) of cases were diagnosed in the first trimester, 45.7% (1,016/2.222) in the second, and 10.8% (240/2,222) in the third trimester.

**Fig. 1:**
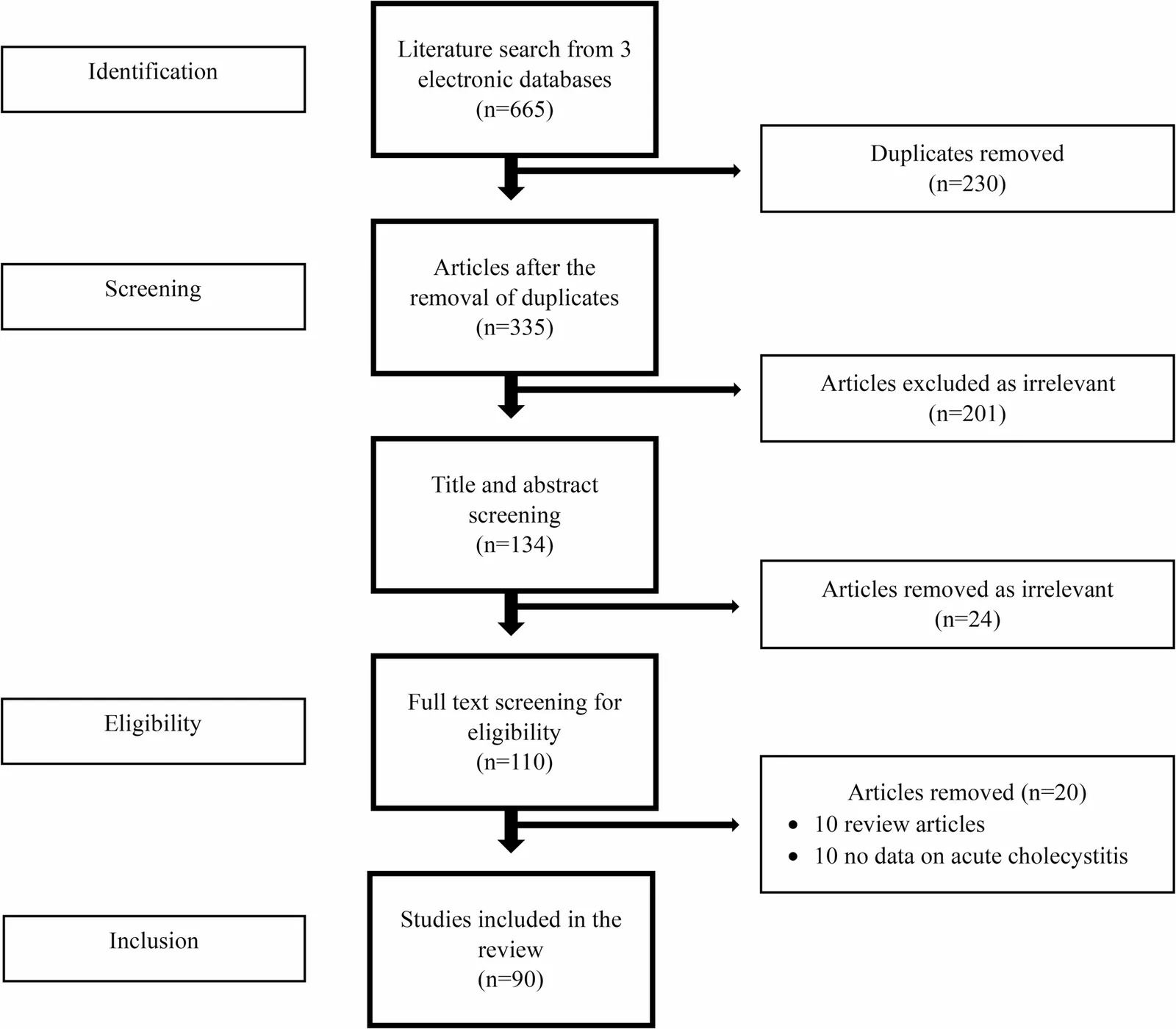
PRISMA flowchart

With regard to management, operative treatment was performed in 224 out of 448 cases (50.0%) where this was reported, while non-operative management was used in 224 out of 448 cases (50.0%). Laparoscopic cholecystectomy performed in 141 of 283 cases (49.8%), while open cholecystectomy was reported in 142 of 283 cases (50.2%). The reported readmission rate was 61 of 240 cases (26.1%), though data were available in only three studies. Complications occurred in 16 of 288 reported cases (5.6%), including a range of minor and major postoperative events. The spontaneous abortion rate was 7.9% (20/254) and antenatal mortality was notably rare, with no recorded events in the studies that reported this outcome (Supplementary Material). (Figure 1).

### Observational studies (operative vs. non operative management)

**Fig. 2:**
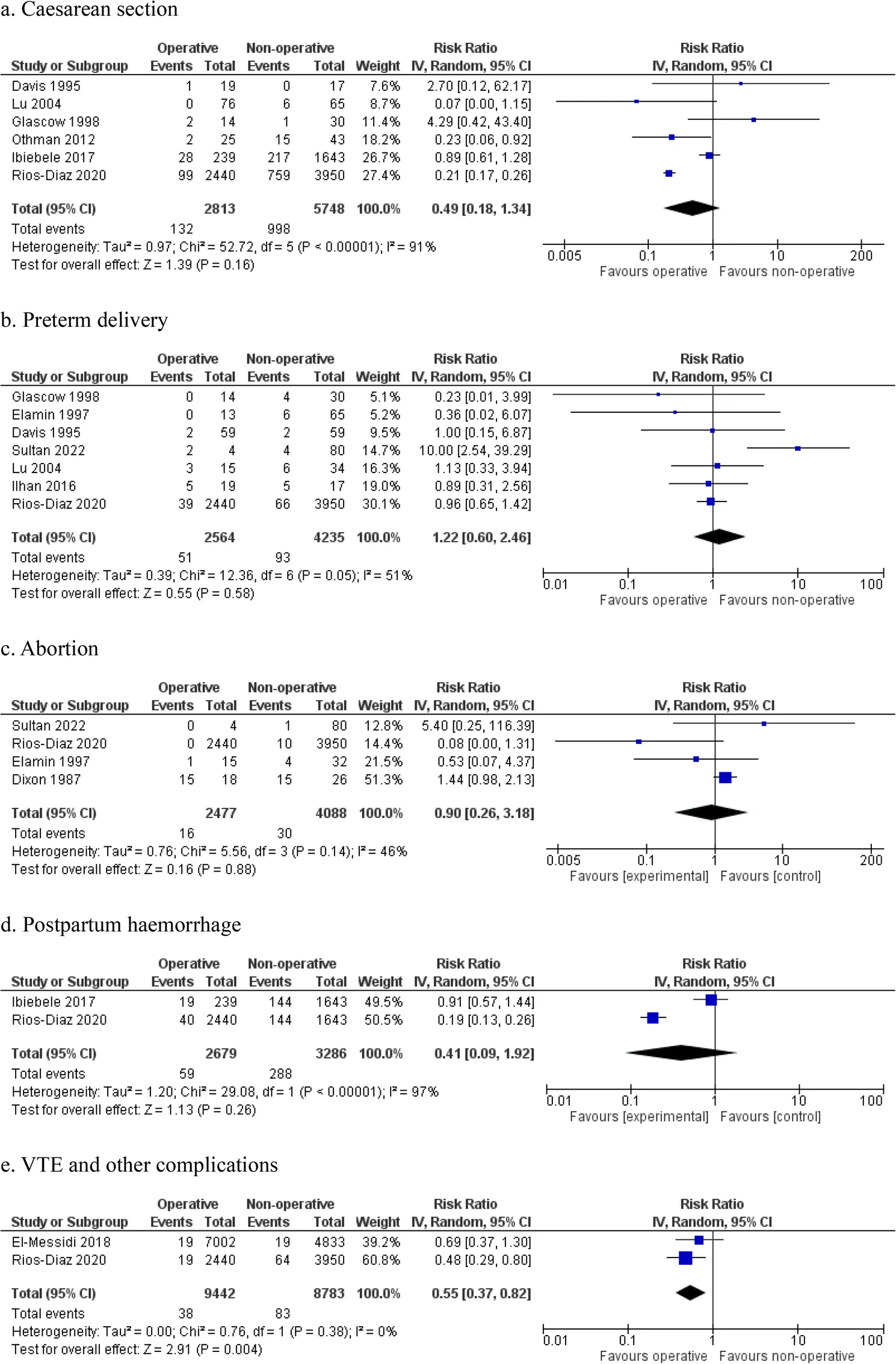
Forrest plots assessing the effect of operative and non-operative management of cholecystitis during pregnancy on the rates of (**a**) caesarean section (**b**) preterm delivery (**c**) abortion (**d**) postpartum haemorrhage (**e**) VTE and other complications. a. Caesarean section b. Preterm delivery

Six comparative studies (*n* = 8,561) reported on mode of delivery, showing a lower proportion of caesarean sections among patients who underwent operative management (132/2,813) compared to those managed non-operatively (998/5,748), though the difference was not statistically significant (RR 0.49, 95% CI 0.18–1.34; I² = 91%, 6 studies; 8,561 participants; very low-certainty evidence; Table 1; Fig. 2, Table S7). Preterm birth was assessed across seven studies (*n* = 6,799), with similar rates between operative (51/2,564) and non-operative groups (93/4,235) (RR 1.22, 95% CI 0.60–2.46; I² = 51%, 7 studies; 6,799 participants; very low-certainty evidence; Table 1; Fig. 2, Table S7).

**Table 1:** Key findings of the included body of evidence

Domain	Finding
Population	90 studies (1964–2023): 48 case reports/series (*n* = 118), 19 descriptive cohorts (*n* = 5,702), 19 comparative studies of operative vs. non-operative (*n* = 91,955), and 4 comparing laparoscopic vs. open (*n* = 62,620).
Symptoms (IPD, *n* = 75 with data)	Right upper-quadrant pain 40%; vomiting 33%; nausea 29%; fever 17%; epigastric pain 9%; chills/myalgias 4%; back pain 1%.
Imaging modality (IPD, *n* = 92 with data)	Gallstones 42% (multiple stones 9%); sonographic cholecystitis/thickened wall 21%; sludge 4%; common bile duct involvement 7%; acalculous 2%; imaging unremarked/not reported 22%.
Therapeutic management (IPD, *n* = 93)	Operative 81 (laparoscopic cholecystectomy 71; percutaneous drainage 10; open cholecystectomy 1); conservative only 12.
Clinical and obstetric outcomes (IPD)	84% (70/83) uncomplicated courses. Among 87 pregnancies: caesarean Sect. 9; preterm delivery 4 (one at 25 weeks); remainder vaginal/uncomplicated or ongoing at time of reporting.
Descriptive cohorts (where reported)	Mean maternal age 28.5 ± 4.6 years; diagnosis most often in 1st–2nd trimester. Management split: operative 224/448 (50%) vs. non-operative 224/448 (50%). Surgical approach: laparoscopic 141/283 (49.8%) vs. open 142/283 (50.2%). Readmission 61/240 (26.1%).

*IPD*: individual patient data

Spontaneous abortion rates were comparable between groups in four studies (RR 0.90, 95% CI 0.26–3.18; I² = 46%, very low-certainty evidence; Table 1; Fig. 2, Table S7), as were post-partum haemorrhage rates, although data were limited to two studies and showed substantial heterogeneity (RR 0.41, 95% CI 0.09–1.92; I² = 97%, 2 studies; 5,965 participants; very low-certainty evidence; Table 1; Fig. 2, Table S7). Notably, venous thromboembolism (VTE) complications were lower in the operative group across two large cohorts (38/9,442 vs. 83/8,783; RR 0.55, 95% CI 0.37–0.82; I² = 0%, 2 studies; 18,225 participants; low-certainty evidence; Table 1; Fig. 2, Table S7). Other complications, reported in eight studies (*n* = 18,824), did not differ significantly between groups (RR 0.96, 95% CI 0.69–1.34; I² = 75%, 8 studies; 18,824 participants; very low-certainty evidence; Table 1; Fig. 2, Table S7) (Table 2; Fig. 2).

**Table 2:** Summary of findings of the primary analysis (operative management vs. non operative management)

Outcome	No of studies	Total No of participants	Operative (events per total *n*/*N* (%))	Non-operative (events per total *n*/*N* (%))	Risk ratio (95% CI) random effects model (inverse variance method)	I^2^	τ^2^
Caesarean Section	6	8561	132/2813	998/5748	0.49 [0.18, 1.34]	91	0.97
Preterm delivery	7	6799	51/2564	93/4235	1.22 [0.60, 2.46]	51	0.39
Abortion	4	6565	16/2477	30/4088	0.90 [0.26, 3.18]	46	0.76
PPH	2	5965	59/2679	288/3286	0.41 [0.09, 1.92]	97	1.2
VTE complications	2	18,225	38/9442	83/8783	0.55 [0.37, 0.82]	0	0
Other complications	8	18,824	516/9723	657/9101	0.96 [0.69, 1.34]	75	0.13

## Discussion

This systematic review and meta-analysis, incorporating data from 90 studies and more than 160,000 pregnancies, provides a comprehensive synthesis to date of acute cholecystitis in pregnancy. We found that right upper quadrant pain, nausea, and vomiting were the most common presenting symptoms, with ultrasound as the primary diagnostic modality. Operative management—predominantly laparoscopic cholecystectomy—emerged as the preferred strategy in reported cases and was generally associated with favourable maternal and neonatal outcomes. Importantly, pooled analyses demonstrated no significant differences between operative and non-operative management with respect to caesarean section, preterm birth, spontaneous abortion, postpartum haemorrhage, or other obstetric complications. Although, operative management conferred a lower risk of venous thromboembolism, this finding was derived from only two studies and should therefore be interpreted cautiously, as differences in thromboprophylaxis practices, patient characteristics, and residual confounding may have contributed to the observed association. Together, these findings reinforce the idea that surgical management, when clinically indicated and after careful patient selection, is safe across pregnancy and may offer benefits over non-operative strategies in selected domains.

### Findings in the context of the literature

A minimally invasive laparoscopic approach is generally favoured over open surgery because of its superior maternal and foetal outcomes, including shorter hospital stays, lower postoperative pain and complication rates, and reduced risk of preterm labour [11–13]. Recent high-quality studies indicate that prompt operative management of acute cholecystitis in pregnancy generally offers better outcomes than non-operative management [10, 14]. A large retrospective cohort of 5,759 pregnant patients comparing those who underwent cholecystectomy versus non-operative treatment found that surgery was associated with significantly lower odds of adverse pregnancy outcomes, including preterm delivery and pregnancy loss, across all trimesters, with the greatest benefit observed in the third trimester [14]. A more recent systematic review and meta-analysis incorporating nine studies (45,883 pregnancies) confirmed these findings: operative treatment significantly reduces a composite of adverse pregnancy outcomes (OR 0.60; 95% CI 0.42 to 0.87) and shortens hospital stay by over seven days, without increasing maternal mortality, pregnancy loss, preterm delivery, or readmission rates [10]. The advantages of operative management likely stem from definitive control of the underlying pathology, prevention of disease progression, and avoidance of recurrent biliary events or unplanned readmissions. In addition, surgical treatment limits prolonged antibiotic exposure and fasting, while laparoscopic techniques confer further benefits such as less postoperative pain, lower wound and respiratory morbidity, and shorter hospitalisation [15–17].

Non-operative management often results in high rates of disease recurrence. Historically, it has been the default approach in pregnancy due to concerns about anaesthetic and surgical risks, but this often resulted in recurrent attacks and frequent readmissions. Percutaneous cholecystostomy has since been explored as a temporising option, particularly in late pregnancy or when surgery is not feasible, though data suggest it may prolong infection and defer rather than resolve the problem [1–3]. Contemporary evidence and guidelines now support early laparoscopic cholecystectomy as the treatment of choice, with non-operative strategies reserved for select scenarios such as patients near term or in centres lacking surgical expertise [18–20]. A nationwide analysis using U.S. data demonstrated that only about 27–35% of pregnant patients received surgery during the initial admission; among those managed non-operatively, around 6% were readmitted for cholecystectomy, and percutaneous cholecystostomy use led to the longest hospital stays [21, 22]. Both the American College of Obstetricians and Gynecologists and the Society of American Gastrointestinal and Endoscopic Surgeons recommend that when surgery is indicated, it should not be delayed solely because of pregnancy, although these recommendations are based on low-certainty evidence derived from observational studies rather than randomised trials [11, 12, 23].

In our review, most cases were identified during the first (43.5%) or second trimester (45.7%), while only 10.8% were diagnosed in the third. This distribution aligns with the long-standing perception that the second trimester has traditionally been considered the safest window for non-obstetric surgery—owing to completed organogenesis, manageable uterine size, and lower miscarriage/preterm birth risks—contemporary data support surgery in any trimester when required [11, 24]. Multiple studies have found no significant increase in maternal or foetal complications when procedures are undertaken in the first or third trimester compared to the second. In fact, postponing a needed operation (especially an emergency) until after the pregnancy can worsen outcomes in some cases [15, 25, 26]. Laparoscopic cholecystectomy has been associated with fewer maternal and fetal complications than open surgery, and national data show that over 95% of such surgeries during pregnancy are done laparoscopically with extremely low serious complication rates [18]. In the third trimester, the increasing size of the uterus and proximity to delivery complicate surgical decisions. Some patients are managed with combined delivery and cholecystectomy during the same admission—either delivery before surgery or, in some cases, concurrent caesarean and cholecystectomy—especially when disease is severe or near term.

Serious complications are rare – modern reports describe minimal maternal mortality and morbidity or foetal loss associated with laparoscopic gallbladder surgery in pregnancy [13, 27]. This body of evidence underpins the current consensus that the timing of surgery should be dictated by clinical necessity rather than trimester, with laparoscopic cholecystectomy as the preferred surgical modality whenever feasible.

### Strengths and limitations

This systematic review and meta-analysis represents a comprehensive synthesis to date of acute cholecystitis during pregnancy, incorporating evidence from over 160,000 pregnancies across multiple study designs and healthcare settings. By analysing individual patient reports separately from larger observational cohorts, we were able to characterise clinical presentation, diagnostic findings, management strategies, and maternal–foetal outcomes, while also performing comparative analyses of operative versus non-operative management.

However, several limitations must be acknowledged. The evidence base consisted predominantly of retrospective and observational studies, introducing potential selection bias, reporting bias, and confounding by indication. In particular, treatment allocation was not randomised, and the decision to pursue operative or non-operative management was influenced by clinical factors that were not consistently reported across studies. Patients undergoing surgery may have differed from non-operatively managed patients in disease severity, symptom burden, gestational age at presentation, comorbidities, and risk profile, which may have influenced the observed outcomes independently of the intervention itself. Randomised controlled trials were unavailable, and case reports often lacked standardised reporting, leading to missing or incomplete data for key variables such as gestational age, operative details, and follow-up duration. Considerable heterogeneity existed between studies in design, patient selection, and outcome definitions, limiting the certainty of pooled estimates despite the use of random-effects models. This heterogeneity was further compounded by the long study period (1964–2023), during which substantial changes occurred in diagnostic imaging, perioperative care, anaesthetic management, and surgical practice, including the transition from open to laparoscopic cholecystectomy. Additionally, the included studies originated from diverse healthcare systems with differing clinical pathways, resource availability, and thresholds for operative intervention, which may have influenced both management decisions and reported outcomes. Furthermore, analyses for some outcomes—such as venous thromboembolism and specific obstetric complications—were based on a small subset of studies, reducing statistical power. Publication bias cannot be excluded, as positive or unusual cases are more likely to be reported than uneventful outcomes.

In addition, trimester-specific analyses were limited by inconsistent reporting of gestational age at diagnosis, timing of intervention and pregnancy outcomes. Disease severity was also variably described, with limited data on complications such as empyema, perforation, pancreatitis, choledocholithiasis or sepsis, making it difficult to fully account for confounding by indication. Important aspects of perioperative and obstetric care, including antibiotic regimens, thromboprophylaxis, fetal monitoring, anaesthetic approach, pneumoperitoneum pressures and use of tocolysis, were rarely reported. Long-term maternal and neonatal outcomes, including recurrent biliary events, readmission, delayed postpartum surgery and infant follow-up, were also poorly captured. These limitations reduce the ability to define optimal patient selection and trimester-specific management strategies.

## Conclusion

This systematic review and meta-analysis provides a comprehensive synthesis to date of acute cholecystitis in pregnancy, integrating evidence from over five decades of global research. The findings indicate that ultrasound-based diagnosis remains the cornerstone of evaluation, and that laparoscopic cholecystectomy appears safe and effective across all trimesters, with obstetric outcomes comparable to non-operative management and a lower risk of thromboembolic events. Although the available evidence is largely observational and heterogeneous, the consistency of results across study designs supports the safety of timely surgical intervention when clinically indicated. Future prospective studies and multicentre registries are needed to refine trimester-specific management strategies, define optimal timing for surgery, and establish standardized outcome reporting to guide evidence-based care.

## Supplementary Information

Supplementary Material 1.

## Data Availability

All data relevant to the study are included in the article or uploaded as supplementary information.

## Abbreviations

IPD: Individual Patient Data
PRISMA: Preferred Reporting Items for Systematic Reviews and Meta-Analyses
MINORS: Methodological Index for Non-Randomized Studies
JBI: Joanna Briggs Institute
VTE: Venous Thromboembolism
PPH: Postpartum Haemorrhage
ERCP: Endoscopic Retrograde Cholangiopancreatography
