# Supplementary material for: Acute cholecystitis during pregnancy: a systematic review and meta-analysis
Source: BMC Surg. 2026 Aug 7;26:748. doi: 10.1186/s12893-026-04087-5 (PMC13737769; doi:10.1186/s12893-026-04087-5)
Supplement: Supplementary file 1 [file 12893_2026_4087_MOESM1_ESM.docx]

**Supplementary material**

**Table S1.** Studies with individual patient data (IPD): Characteristics of the included studies.

| **Author**  **Country Year** | **Case,**  **Age** | **GP,**  **GW** | **Presenting Symptoms** | **Management** | **Obstetric outcomes** | |
| --- | --- | --- | --- | --- | --- | --- |
|  |  |  |  |  | **Mode of Delivery (neonatal outcome)** | **GA at delivery** |
| Spinapolice  USA  1982 | Case 1, 29 | G7P2,  27 weeks | - fever, - nausea, - vomiting, - RUQ pain radiating to right costovertebral angle | - Operative: N/A, - Non-operative: Abx   (27 weeks) | (healthy infant) | N/A |
| Lewis  USA  1983 | Case 1, 40 | G7P6,  28 weeks | - epigastric pain, - vomiting | - Operative: Laparotomy, - Non-operative: IVF, IV Abx   (30 weeks) | Vaginal Delivery (healthy infant) | 36 weeks |
| Weber  USA  1991 | Case 1, 41 | G8P3,  13.5 weeks | - worsening RUQ pain, - nausea, - vomiting | - Operative: N/A, - Non-operative: N/A   (13.5 weeks) | N/A | N/A |
| Soper  USA  1992 | Case 1, 20 | G1P0,  19 weeks | - RUQ discomfort, - 5kg weight loss over 2 months | - Operative: Laparoscopic cholecystectomy, - Non-operative: progesterone 250 mg IM, magnesium sulphate drip IV   (19 weeks) | Spontaneous vaginal delivery (healthy infant) | 40 weeks |
|  | Case 2, 25 | N/A,  15 weeks | - RUQ pain, - nausea, - vomiting | - Operative: Laparoscopic cholecystectomy, - Non-operative: magnesium sulphate drip IV   (15 weeks) | Spontaneous vaginal delivery (healthy infant) | 40 weeks |
|  | Case 3, 26 | N/A,  15 weeks | - severe biliary colic, - RUQ pain | - Operative: Laparoscopic cholecystectomy, - Non-operative: N/A   (15 weeks) | Spontaneous vaginal delivery (healthy infant) | 39 weeks |
|  | Case 4, 22 | N/A,  13 weeks | - abdominal pain with each meal, - weight loss | - Operative: Laparoscopic cholecystectomy, - Non-operative: N/A   (13 weeks) | Pregnancy ongoing | |
|  | Case 5, 42 | N/A,  14 weeks | - recurring RUQ & back pain postprandially, - weight loss | - Operative: Laparoscopic cholecystectomy, - Non-operative: N/A   (14 weeks) | Pregnancy ongoing | |
| Bennett  USA  1993 | Case 1, 30 | G3P2,  19 weeks | - acute RUQ pain, - nausea, - vomiting | - Operative: Laparoscopic cholecystectomy, - Non-operative: N/A   (19 weeks) | Vaginal delivery (healthy infant) | 39 weeks |
| Costantino  USA  1994 | Case 1, 23 | G3P2,  10 weeks | - RUQ pain, - nausea, - vomiting, - losing weight | - Operative: laparoscopic cholecystectomy, - Non-operative: dehydration and pain management   (14 weeks) | Vaginal delivery (healthy infant) | 40 weeks |
|  | Case 2, 26 | G2P1,  19 weeks | - multiple attacks of right upper quadrant abdominal pain | - Operative: Laparoscopic cholecystectomy, - Non-operative: N/A   (22 weeks) | Vaginal delivery (healthy infant) | 40 weeks |
| Chandra  USA  1994 | Case 1, 28 | N/A,  13 weeks | - Intermittent RUQ pain | - Operative: Laparoscopic cholecystectomy, - Non-operative: N/A   (13 weeks) | N/A |  |
| Shaked  Israel  1994 | Case 1, 34 | N/A,  10.5 weeks | - epigastric pain, - vomiting | - Operative: Laparoscopic cholecystectomy, - Non-operative: IV fluids, Abx, tocolytic agent   (10.5 weeks) | Vaginal delivery (healthy infant) | 40 weeks |
| Allmendinger  USA  1995 | Case 1, 33 | G5P3,  30 weeks | - RUQ pain, - nausea, - vomiting | - Operative: ultrasound guided percutaneous transhepatic cholecystostomy, - Non-operative: Abx   (30 weeks) | Vaginal delivery (healthy infant) | 41 weeks |
|  | Case 2, 23 | G1P0,  32 weeks | - RUQ & back & epigastric pain - vomiting | - Operative: ultrasound guided percutaneous transhepatic cholecystostomy, - Non-operative: Abx   (32 weeks) | C-section | 33 weeks |
| Iafrati  USA  1995 | Case 1, 38 | G2P1,  14 weeks | - RUQ pain and nausea - vomiting - fever | - Operative: Gasless laparoscopic cholecystectomy, - Non-operative: IV fluids, Abx   (14 weeks) | N/A | N/A |
| Lanzafame USA  1995 | Case 1, 33 | G2P1,  17 weeks | - daily RUQ pain | - Operative: Laparoscopic cholecystectomy, - Non-operative: N/A   (17 weeks) | N/A | N/A |
|  | Case 2, 33 | G4P3,  22 weeks | - daily RUQ pain, - weight loss | - Operative: Laparoscopic cholecystectomy, - Non-operative: N/A   (22 weeks) | N/A | N/A |
|  | Case 3, 28 | G2P1,  27.5 weeks | - daily RUQ pain, - weight loss | - Operative: Laparoscopic cholecystectomy, - Non-operative: N/A   (27.5 weeks) | N/A | N/A |
|  | Case 4, 31 | G2P1,  28 weeks | - biliary colic, - daily RUQ pain | - Operative: Laparoscopic cholecystectomy, - Non-operative: N/A   (28 weeks) | N/A | N/A |
|  | Case 5, 33 | G3P2,  14 weeks | - N/A | - Operative: Laparoscopic cholecystectomy, - Non-operative: N/A   (14 weeks) | N/A | N/A |
| Andreoli USA  1996 | Case 1, 23 | G3P1,  26 weeks | - severe epigastric pain, - nausea, - nonbilious vomiting | - Operative: Laparoscopic cholecystectomy, - Non-operative: fasting, IV fluids   (28 weeks) | Vaginal delivery (healthy infant) | 39 weeks |
| Grimes  USA  1996 | Case 1, 36 | G5P3,  10 weeks | - periumbilical pain radiating to RLQ | - Operative: Exploratory laparotomy cholecystectomy, appendicectomy, - Non-operative: Abx   (10 weeks) | Vaginal delivery (healthy infant) | 40 weeks |
| Abuabara USA  1997 | Case 1, 24 | G4P3,  21 weeks | - Pain, - nausea, - vomiting | - Operative: Laparoscopic cholecystectomy, - Non-operative: NBM, IV fluids   (21 weeks) | (healthy infant) | N/A |
|  | Case 2, 23 | G3P2,  5 weeks | - Pain, - nausea, - vomiting, - fever | - Operative: Laparoscopic cholecystectomy, trancystic common bile duct exploration, - Non-operative: NBM, IV fluids   (5 weeks) | Pregnancy ongoing | |
|  | Case 3, 19 | G1P0,  30 weeks | - Pain, - nausea, - vomiting, - fever | - Operative: Laparoscopic cholecystectomy, - Non-operative: NBM, IV fluids   (30 weeks) | (healthy infant) | N/A |
|  | Case 4, 23 | G3P2,  31 weeks | - Pain, - nausea, - vomiting | - Operative: Laparoscopic cholecystectomy, - Non-operative: NBM, IV fluids   (31 weeks) | (healthy infant) | N/A |
|  | Case 5, 27 | G2P1,  19 weeks | - Pain, - nausea, - vomiting | - Operative: Laparoscopic cholecystectomy, - Non-operative: NBM, IV fluids   (19 weeks) | (healthy infant) | N/A |
|  | Case 6, 19 | G3P1,  18 weeks | - Pain, - nausea, - vomiting | - Operative: Laparoscopic cholecystectomy, - Non-operative: NBM, IV fluids   (18 weeks) | (healthy infant) | N/A |
|  | Case 7, 24 | G3P2,  20 weeks | - Pain, - nausea, - vomiting | - Operative: Laparoscopic cholecystectomy, - Non-operative: NBM, IV fluids   (20 weeks) | (healthy infant) | N/A |
|  | Case 8, 21 | G2P1,  19 weeks | - Pain, - nausea, - vomiting | - Operative: Laparoscopic cholecystectomy, - Non-operative: NBM, IV fluids   (19 weeks) | (healthy infant) | N/A |
|  | Case 9, 23 | G3P2,  28 weeks | - Pain, - nausea, - vomiting | - Operative: Laparoscopic cholecystectomy, - Non-operative: NBM, IV fluids   (28 weeks) | (healthy infant) | N/A |
|  | Case 10, 21 | G2P1,  22 weeks | - Pain, - nausea, - vomiting | - Operative: Laparoscopic cholecystectomy, - Non-operative: NBM, IV fluids   (22 weeks) | (healthy infant) | N/A |
|  | Case 11, 22 | G1P0,  21 weeks | - Pain, - nausea, - vomiting | - Operative: Laparoscopic cholecystectomy, - Non-operative: NBM, IV fluids   (21 weeks) | (healthy infant) | N/A |
|  | Case 12, 18 | G2P1,  29 weeks | - Pain, - nausea, - vomiting | - Operative: Laparoscopic cholecystectomy, - Non-operative: NBM, IV fluids   (29 weeks) | (healthy infant) | N/A |
|  | Case 13, 20 | G2P0,  22 weeks | - Pain, - nausea, - vomiting | - Operative: Laparoscopic cholecystectomy, - Non-operative: NBM, IV fluids   (22 weeks) | (healthy infant) | N/A |
|  | Case 14, 23 | G3P1,  8 weeks | - Pain, - nausea, - vomiting | - Operative: Laparoscopic cholecystectomy, - Non-operative: NBM, IV fluids   (8 weeks) | Pregnancy ongoing | |
|  | Case 15, 21 | G2P1,  19 weeks | - Pain, - nausea, - vomiting, - fever | - Operative: Laparoscopic cholecystectomy, - Non-operative: NBM, IV fluids   (19 weeks) | (healthy infant) | N/A |
|  | Case 16, 25 | G2P1,  15 weeks | - Pain, - nausea, - vomiting | - Operative: Laparoscopic cholecystectomy, - Non-operative: NBM, IV fluids   (15 weeks) | (healthy infant) | N/A |
|  | Case 17, 22 | G5P5,  25 weeks | - Pain, - nausea, - vomiting | - Operative: Laparoscopic cholecystectomy, trancystic common bile duct exploration, - Non-operative: NBM, IV fluids   (25 weeks) | (healthy infant) | 29 weeks |
|  | Case 18, 18 | G1P0,  16 weeks | - Pain, - nausea, - vomiting | - Operative: Laparoscopic cholecystectomy, - Non-operative: NBM, IV fluids   (16 weeks) | (healthy infant) | N/A |
|  | Case 19, 21 | G1P0,  19 weeks | - Pain, - nausea, - vomiting | - Operative: Laparoscopic cholecystectomy, - Non-operative: NBM, IV fluids   (19 weeks) | (healthy infant) | N/A |
|  | Case 20, 17 | G2P1,  14 weeks | - Pain, - nausea, - vomiting | - Operative: Laparoscopic cholecystectomy, - Non-operative: NBM, IV fluids   (14 weeks) | (healthy infant) | N/A |
|  | Case 21, 29 | G3P2,  11 weeks | - Pain, - nausea, - vomiting | - Operative: Laparoscopic cholecystectomy, - Non-operative: NBM, IV fluids   (11 weeks) | (healthy infant) | N/A |
|  | Case 22, 31 | G2P0,  17 weeks | - Pain, - nausea, - vomiting | - Operative: Laparoscopic cholecystectomy, - Non-operative: NBM, IV fluids   (17 weeks) | (healthy infant) | N/A |
| Thomas  US  1998 | Case 1, 23 | N/A,  14 weeks | - N/A | - Operative: Laparoscopic cholecystectomy, - Non-operative: N/A   (14 weeks) | (healthy infant) | N/A |
|  | Case 2, 37 | N/A,  16 weeks | - N/A | - Operative: Laparoscopic cholecystectomy, - Non-operative: N/A   (16 weeks) | (healthy infant) | N/A |
|  | Case 3, 22 | N/A,  10 weeks | - N/A | - Operative: Laparoscopic cholecystectomy, - Non-operative: N/A   (10 weeks) | Elective termination Of pregnancy | |
|  | Case 4, 24 | N/A,  30 weeks | - N/A | - Operative: Laparoscopic cholecystectomy, - Non-operative: N/A   (30 weeks) | Premature delivery (healthy infant) | 36 weeks |
| Coelho  Brazil  1999 | Case 1, 34 | N/A,  29 weeks | - right hypochondrial & epigastric pain, - nausea, - vomiting | - Operative: Laparoscopic cholecystectomy, - Non-operative: NBM, IV fluids, antispasmodics   (29 weeks) | N/A | N/A |
|  | Case 2, 32 | N/A,  30 weeks | - upper abdominal pain, - nausea, - vomiting | - Operative: Laparoscopic cholecystectomy, - Non-operative: IV fluids, analgesic, antispasmodic medication indicated   (30 weeks) | N/A | N/A |
| Grigoriu Brazil  2003 | Case 1, 26 | N/A,  9.5 weeks | - right hypochondrial & epigastric pain, - nausea, - vomiting | - Operative: Laparoscopic cholecystectomy, - Non-operative: IV Abx, LMWH, antispasmodic medication   (9.5 weeks) | N/A | N/A |
|  | Case 2, 30 | N/A,  20.5 weeks | - right hypochondrial & epigastric pain, - nausea, - vomiting | - Operative: Laparoscopic cholecystectomy, - Non-operative: N/A   (20.5 weeks) | N/A | N/A |
| Rizzo  USA  2003 | Case 1, 24 | N/A,  12 weeks | - N/A | - Operative: Laparoscopic cholecystectomy, - Non-operative: N/A   (12 weeks) | Vaginal delivery (healthy infant) | 39 weeks |
|  | Case 2, 23 | N/A,  20 weeks | - N/A | - Operative: Laparoscopic cholecystectomy, - Non-operative: N/A   (20 weeks) | Vaginal delivery (healthy infant) | 37 weeks |
|  | Case 3, 38 | N/A,  17 weeks | - N/A | - Operative: Laparoscopic cholecystectomy, - Non-operative: N/A   (17 weeks) | Vaginal delivery (healthy infant) | 44 weeks |
|  | Case 4, 29 | N/A,  10 weeks | - N/A | - Operative: Laparoscopic cholecystectomy, - Non-operative: N/A   (10 weeks) | Vaginal delivery (healthy infant) | 38 weeks |
|  | Case 5, 32 | N/A,  27 weeks | - N/A | - Operative: Laparoscopic cholecystectomy, - Non-operative: N/A   (27 weeks) | Vaginal delivery (healthy infant) | 40 weeks |
| Machado USA  2004 | Case 1, 36 | G6P4,  26 weeks | - acute RUQ pain, - nausea, - vomiting, - low grade fever | - Operative: Laparoscopic cholecystectomy, - Non-operative: IV fluids, IV Abx   (26 weeks) | Vaginal delivery | 40 weeks |
| Kamitani  Japan  2006 | Case 1, 20 | N/A,  12 weeks | - epigastric pain | - Operative: Laparoscopic cholecystectomy, - Non-operative: N/A   (12 weeks) | N/A | N/A |
| Syed  UK  2007 | Case 1, 18 | N/A,  32 weeks | - acute upper abdominal pain, - vomiting | - Operative: Laparoscopic cholecystectomy, - Non-operative: N/A   (32 weeks) | Vaginal delivery (healthy infant) | 40 weeks |
| Berrington USA  2007 | Case 1, 29 | G1P0,  13 weeks | - fevers, - abdominal pain, - chills, - severe myalgias, - arthralgias, - diarrhoea | - Operative: N/A, - Non-operative: Antibiotics   (13 weeks) | Vaginal delivery (healthy infant) | 40 weeks |
| Başaran Turkey  2007 | Case 1, 30 | G6P0,  22 weeks | - lower quadrant tenderness, - fever, - RUQ pain, - uterine contractions | - Operative: Appendectomy, Cholecystectomy, - Non-operative: N/A   (22 weeks) | Vaginal delivery (healthy infant) | 40 weeks |
| Meshikhes, Saudi Arabia, 2008 | Case 1, 35 | N/A,  32 weeks | - severe colicky RUQ pain, - vomiting | - Operative: Laparoscopic cholecystectomy, - Non-operative: conservative management   (32 weeks) | Vaginal delivery (healthy infant) | 40 weeks |
| Tseng  Taiwan  2009 | Case 1, 41 | G2P0,  19 weeks | - upper abdominal pain | - Operative: ultrasound-guided percutaneous transhepatic placement of pigtail drainage system into gallbladder (PTGBD), - Non-operative: N/A   (19 weeks) | N/A | N/A |
| Machado Oman  2009 | Case 1, 22 | N/A,  26 weeks | - N/A | - Operative: Laparoscopic cholecystectomy, - Non-operative: indomethacin (tocolytic agent)   (26 weeks) | Spontaneous vaginal delivery (healthy infant) | 40 weeks |
|  | Case 2, 23 | N/A,  26 weeks | - N/A | - Operative: Laparoscopic cholecystectomy, - Non-operative: indomethacin (tocolytic agent)   (26 weeks) | Spontaneous vaginal delivery (healthy infant) | 40 weeks |
|  | Case 3, 25 | N/A,  28 weeks | - N/A | - Operative: Laparoscopic cholecystectomy, - Non-operative: N/A   (28 weeks) | Spontaneous vaginal delivery (healthy infant) | 40 weeks |
| RavichandranIndia  2009 | Case 1, 29 | G1P0,  13 weeks | - fever, - abdominal pain with chills, - severe myalgia, - arthralgia | - Operative: nil, - Non-operative: platelet transfusion, antibiotic therapy (ampicillin, gentamicin, azithromycin)   (13 weeks) | Spontaneous vaginal delivery (healthy infant) | 40 weeks |
| Marasinghe Sri lanka 2011 | Case 1, 29 | G2P1,  29 weeks | - fever, - right hypochondrial pain, - backache, - fatigue | - Operative: N/A, - Non-operative: Abx, IVF   (29 weeks) | Vaginal delivery (healthy infant) | 40 weeks |
| Augustin Croatia  2012 | Case 1, 33 | G1P0,  16 weeks | - RUQ pain | - Operative: Laparoscopic cholecystectomy, - Non-operative: N/A   (16 weeks) | Elective C-section (healthy infant) | 39 weeks |
| Kocum  Croatia  2012 | Case 1, 26 | N/A,  13 weeks | - N/A | - Operative: Laparoscopic cholecystectomy, - Non-operative: N/A   (13 weeks) | Vaginal delivery at 40 weeks | 40 weeks |
| Pezzolla Croatia  2012 | Case 1, 36 | N/A,  11.4 weeks | - upper abdominal pain, - nausea, - vomiting | - Operative: Laparoscopic cholecystectomy, - Non-operative: N/A   (11.4 weeks) | N/A | N/A |
| Sucandy  USA  2013 | Case 1, 29 | N/A,  5 weeks | - N/A | - Operative: ERCP, - Non-operative: N/A   (5 weeks) | N/A | N/A |
| Ibitoye  USA  2014 | Case 1, 32 | N/A,  N/A | - N/A | - Operative: Laparoscopic cholecystectomy, - Non-operative: conservative management | N/A | N/A |
|  | Case 2, 33 | N/A,  N/A | - N/A | - Operative: N/A, - Non-operative: conservative management | N/A | N/A |
|  | Case 3, 34 | N/A,  N/A | - N/A | - Operative: N/A, - Non-operative: conservative management | N/A | N/A |
| Kocael  USA  2015 | Case 1, 28 | N/A,  18 weeks | - RUQ pain, - nausea, - vomiting, - T 37.2 | - Operative: Laparoscopic cholecystectomy, - Non-operative: Abx, prophylactic tocolysis   (18 weeks) | Premature delivery (healthy infant) | 35 weeks |
|  | Case 2, 25 | N/A,  15 weeks | - RUQ pain, - nausea, - vomiting, - T 37.5 | - Operative: Laparoscopic cholecystectomy, - Non-operative: Abx   (15 weeks) | (healthy infant) | 37 weeks |
| Eller Denmark 2016 | Case 1, 35 | G1P0,  36 weeks | - RUQ pain and discomfort, - vomiting | - Operative: Percutaneous ultrasound-guided transhepatic cholecystostomy (PUTC), - Non-operative: metronidazole, cefuroxime   (36 weeks) | C-section  (healthy infant) | 39 weeks |
| Kosmidis Greece  2016 | Case 1, 33 | G2P1,  16 weeks | - acute abdomen, - nausea, - fever | - Operative: 2nd adm diagnostic laparoscopy, Laparoscopic cholecystectomy and adhesiolysis, - Non-operative: 1st adm bowel rest, IVF, analgesics, colchicine/2nd adm bowel rest, hydration, Cefuroxime, Metronidazole, analgesics, colchicine   (17 weeks) | Vaginal delivery | 40 weeks |
| Caliskan Turkey  2017 | Case 1, 36 | G2P0,  6 weeks | - RUQ pain, - nausea, - vomiting | - Operative: Percutaneous cholecystostomy, Laparoscopic cholecystectomy, - Non-operative: NBM, IV fluids, Abx   (6 weeks) | CS, LBWI at 32 weeks | 32 weeks |
|  | Case 2, 29 | G2P2,  34 weeks | - RUQ pain, - nausea, - vomiting | - Operative: Percutaneous cholecystectomy, Laparoscopic cholecystectomy, - Non-operative: NBM, IV fluids, Abx   (34 weeks) | C-section | N/A |
|  | Case 3, 31 | G1P0,  10 weeks | - RUQ pain, - nausea, - vomiting | - Operative: Percutaneous cholecystectomy, Laparoscopic cholecystectomy, - Non-operative: NBM, IV fluids, Abx   (10 weeks) | C-section | N/A |
|  | Case 4, 31 | G3P1,  32 weeks | - RUQ pain, - nausea, - vomiting | - Operative: Percutaneous cholecystectomy, Laparoscopic cholecystectomy, - Non-operative: NBM, IV fluids, Abx   (32 weeks) | C-section | N/A |
|  | Case 5, 32 | G3P2,  35 weeks | - RUQ pain, - nausea, - vomiting | - Operative: Percutaneous cholecystectomy, Laparoscopic cholecystectomy, - Non-operative: NBM, IV fluids, Abx   (35 weeks) | Normal vaginal delivery | N/A |
|  | Case 6, 22 | G3P2,  30 weeks | - RUQ pain, - nausea, - vomiting | - Operative: Laparoscopic cholecystectomy, - Non-operative: NBM, IV fluids, Abx   (30 weeks) | CS | N/A |
| Mosawi  Iran  2019 | Case 1, 17 | N/A,  22 weeks | - RUQ pain, - nausea, - vomiting, - anorexia | - Operative: Ascaris worms were removed, CBD exploration, several lavage, - Non-operative: N/A (22 weeks) | N/A | N/A |
| Qiu  China  2019 | Case 1, 38 | N/A,  8 weeks | - acute onset RUQ pain | - Operative: Laparoscopic cholecystectomy, - Non-operative: N/A   (8 weeks) | Vaginal delivery (healthy infant) | 40 weeks |
| Handaya Indonesia 2021 | Case 1, 37 | N/A,  22 weeks | - fever, - diffuse abdominal pain, - SOB | - Operative: open cholecystectomy with subcostal (Kocher) incision, - Non-operative: N/A   (22 weeks) | Vaginal delivery (healthy infant) | 40 weeks |
| Ward  USA  2021 | Case 1, 22 | G3P2,  32 weeks | - constant RUQ pain | - Operative: Laparoscopic cholecystectomy, - Non-operative: N/A   (32 weeks) | Vaginal delivery (healthy infant) | 40 weeks |
| Mokhtari USA  2021 | Case 1, 16 | G1P0,  35 weeks | - fever, - myalgias, - SOB, - dry cough, - tachypnoeic | - Operative: N/A, - Non-operative: Antibiotics, 30 L/min of high flownasal cannula at 50% FiO2   (35 weeks) | Vaginal delivery (healthy infant) | 40 weeks |
| Hojberg  USA  2022 | Case 1, 41 | G5P4,  34 weeks | - epigastric & RUQ & middle back pain | - Operative: Percutaneous Cholecystostomy Tube, - Non-operative: N/A   (34 weeks) | C-section  (breech-breech twins) | 37 weeks |

**Table 2.** Descriptive cohorts: Characteristics of the included studies.

| **Author Country Year** | **Study Design** | **No of patient with cholecystitis** | **Mean Age** | **Timing of Diagnosis** | | | **Management** | |
| --- | --- | --- | --- | --- | --- | --- | --- | --- |
|  |  |  |  | **1^st^ Trimester** | **2^nd^ Trimester** | **3^rd^ Trimester** | **Operative** | **Non-operative** |
| Greene,  Canada,  1963 | retrospective cohort | 17 | 26.5 | 8/17 | 7/17 | 2/17 | N/A | N/A |
| Hiatt,  USA,  1986 | retrospective cohort | 22 | 27 | 7/26 | 5/26 | 12/26 | 19/26 | 7/26 |
| Erelding,  USA,  1993 | retrospective cohort | 5 | N/A | 1/5 | 3/5 | 1/5 | N/A | N/A |
| El Masry,  Saudi Arabia,  1997 | retrospective cohort | 3 | 29.6 | N/A | N/A | N/A | N/A | N/A |
| Reyes-Tineo,  Puerto Rico,  1997 | retrospective cohort | 5 | 24.4 | 1/5 | 1/5 | 3/5 | 5/5 | N/A |
| Cosenza,  USA,  1999 | retrospective cohort | 12 | 29 | 3/12 | 8/12 | 1/12 | 32/69 | 37/69 |
| Sungler,  Austria,  2000 | retrospective cohort | 37 | N/A | N/A | 4/5 | 1/5 | N/A | N/A |
| Chiedozi,  Saudi Arabia,  2001 | retrospective cohort | 48 | 39.2 | 82/162 | 30/162 | 50/162 | 14/162 | 148/162 |
| Al-Akeely,  Saudi Arabia,  2003 | retrospective cohort | 32 | 32 | 3/32 | 10/32 | 19/32 | 10/32 | 22/32 |
| Merabishvili,  Georgia,  2007 | case control (abstract) | 30 | N/A | N/A | N/A | N/A | N/A | N/A |
| Acs,  Hungary,  2009 | case control | 254 | 27.9 | N/A | N/A | N/A | N/A | N/A |
| Chiappetta Porras, Argentina,  2009 | prospective cohort | 51 | 24.6 | N/A | N/A | N/A | N/A | N/A |
| Veerappan,  USA,  2013 | retrospective cohort | 7 | 27.2 | 11/56 | 24/56 | 21/56 | N/A | N/A |
| del Avila-Silva,  Mexico,  2014 | retrospective cohort | 20 | N/A | N/A | N/A | N/A | N/A | N/A |
| Paramanathan, Australia,  2015 | retrospective cohort | 22 | N/A | 2/22 | 18/22 | 2/22 | 12/22 | 10/22 |
| Weiner,  Israel,  2015 | retrospective cohort | 7 | 29.2 | N/A | N/A | N/A | N/A | N/A |
| Kolbeinsson,  Iceland,  2016 | retrospective cohort | 7 | N/A | N/A | N/A | N/A | N/A | N/A |
| Alvarez Villasenor,  Mexico,  2016 | retrospective cohort | 33 | 22 | N/A | N/A | N/A | 33/33 | 0/33 |
| Hantouli,  USA,  2023 | retrospective cohort | 5,123 | N/A | 848/1885 | 912/1885 | 291/1885 | N/A | N/A |

**Table S3.** Operative against non-operative management: Characteristics of included studies.

| **Author**  **Country**  **Year** | **Study design** | **No of cholecystitis cases** | **No of patients** | | **Age (mean/median)** | |
| --- | --- | --- | --- | --- | --- | --- |
|  |  |  | **Operative** | **Non-operative** | **Operative** | **Non-operative** |
| Dixon  USA  1987 | retrospective cohort | 44 | 18 | 26 | 26.7 (mean) | 26 (mean) |
| Landers  USA  1987 | retrospective cohort | 30 | n/a | 21 | 26.1 (mean) | 26.1 (mean) |
| Morrell  USA  1992 | retrospective cohort | 5 | n/a | n/a | n/a | n/a |
| Swisher  USA  1994 | retrospective cohort | 9 | n/a | n/a | 26.9 (mean) | 26.9 (mean) |
| Davis  USA  1995 | retrospective cohort | 42 | 19 | 17 | n/a | n/a |
| Elamin  Saudi Arabia  1997 | retrospective cohort | 49 | 15 | 34 | n/a | n/a |
| Glascow  USA  1998 | retrospective cohort | 45 | 14 | 30 | 28.3 (mean) | n/a |
| Lu  USA  2004 | retrospective cohort | 76 | 13 | 65 | n/a | n/a |
| Dhupar  USA  2010 | retrospective cohort | 58 | 19 | 39 | n/a | n/a |
| Othman  USA  2012 | retrospective cohort | 112 | 44 | 68 | 25 (mean) | 25 (mean) |
| Jorge  USA  2015 | retrospective cohort | 53 | n/a | n/a | 34 (median) | 31 (median |
| İlhan  Turkey  2016 | retrospective cohort | 59 | 15 | 44 | n/a | n/a |
| Ibiebele  Australia  2017 | retrospective cohort | 1882 | 239 | 1634 | n/a | n/a |
| Hedström  Sweden  2017 | retrospective cohort | 22 | 35 | 62 | 29 (mean) | 32 (mean) |
| El-Messidi  Canada  2018 | retrospective cohort | 59175 | 7002 | 4833 | n/a | n/a |
| Barut  Turkey  2019 | retrospective cohort | 20 | n/a | n/a | 29.5 (mean) | 29.5 (mean) |
| Rios-Diaz  USA  2020 | retrospective cohort | 6390 | 2440 | 3950 | 26.83 (mean) | 27.14 (mean) |
| Cheng  USA  2020 | retrospective cohort | 23939 | n/a | n/a | 26 (median) | 26 (median) |
| Sultan  Iraq  2022 | retrospective cohort | 84 | 4 | 80 | n/a | n/a |

**Table S4.** Studies reporting on open versus laparoscopic management: Characteristics of included studies.

| **Author**  **Country**  **Year** | **Study design** | **No of cholecystitis cases** | **No of patients** | | **Age (mean/median)** | |
| --- | --- | --- | --- | --- | --- | --- |
|  |  |  | **Laparoscopic** | **Open** | **Laparoscopic** | **Open** |
| Cheng  USA  2020 | retrospective cohort | 23,939 | 14259 | 1002 | 25 (median) | 27 (median) |
| Shigemi  Japan  2019 | retrospective cohort | 6018 | n/a | n/a | n/a | n/a |
| Cox  USA  2016 | retrospective cohort | 664 | 606 | 58 | 28.3 (mean) | 28.7 (mean) |
| Barone  USA  1999 | retrospective cohort | 46 | 20 | 26 | 25.4 (mean) | 24.8 (mean) |

**Table S6.** Risk of bias assessment for case reports and series (Joanna Briggs institute tool).

| **Author, year** | **Q1** | **Q2** | **Q3** | **Q4** | **Q5** | **Q6** | **Q7** | **Q8** |
| --- | --- | --- | --- | --- | --- | --- | --- | --- |
| Hojberg, 2022 | ● | ● | ● | ● | ● | ● | ● | ● |
| Mokhtari, 2021 | ● | ● | ᴓ | ○ | ᴓ | ● | ● | ● |
| Ward, 2021 | ● | ○ | ● | ● | ● | ● | ◌ | ● |
| Yuda Handaya, 2021 | ○ | ● | ● | ● | ● | ● | ● | ● |
| Mosawi, 2019 | ○ | ● | ○ | ● | ● | ○ | ◌ | ● |
| Qiu, 2019 | ᴓ | ○ | ○ | ● | ● | ● | ◌ | ● |
| Eller, 2016 | ● | ● | ○ | ᴓ | ● | ● | ◌ | ● |
| Kosmidis, 2016 | ● | ● | ● | ● | ● | ● | ◌ | ● |
| Augustin, 2012 | ● | ○ | ○ | ● | ● | ● | ◌ | ● |
| Kocum, 2012 | ● | ᴓ | ○ | ○ | ● | ● | ◌ | ● |
| Pezzolla, 2012 | ᴓ | ● | ● | ● | ● | ● | ◌ | ● |
| Marasinghe, 2011 | ○ | ● | ● | ● | ● | ● | ◌ | ● |
| Tseng, 2009 | ● | ● | ᴓ | ● | ● | ● | ◌ | ● |
| Meshikhes, 2008 | ● | ᴓ | ● | ● | ● | ● | ◌ | ● |
| Başaran, 2007 | ● | ● | ○ | ● | ○ | ○ | ◌ | ᴓ |
| Berrington, 2007 | ● | ● | ● | ● | ● | ● | ◌ | ● |
| Merabishvili, 2007 | ● | ● | ○ | ● | ○ | ○ | ◌ | ᴓ |
| Rangarajan, 2007 | ᴓ | ○ | ● | ᴓ | ● | ● | ◌ | ● |
| Syed, 2007 | ᴓ | ● | ● | ● | ● | ● | ◌ | ● |
| Kamitani, 2006 | ᴓ | ● | ᴓ | ○ | ● | ● | ◌ | ● |
| Grigoriu, 2004 | ᴓ | ● | ᴓ | ᴓ | ● | ᴓ | ◌ | ● |
| Machado, 2004 | ● | ● | ● | ● | ᴓ | ● | ◌ | ● |
| Coelho, 1999 | ᴓ | ○ | ● | ● | ᴓ | ● | ◌ | ● |
| Thomas, 1998 | ● | ○ | ○ | ○ | ● | ○ | ◌ | ● |
| Andreoli, 1996 | ● | ○ | ● | ● | ● | ● | ● | ● |
| Grimes, 1996 | ● | ○ | ● | ᴓ | ● | ● | ◌ | ● |
| Allmendiger, 1995 | ● | ● | ● | ● | ● | ● | ◌ | ● |
| Chandra, 1994 | ● | ○ | ● | ᴓ | ● | ● | ◌ | ● |
| Costantino, 1994 | ● | ○ | ● | ○ | ● | ● | ◌ | ● |
| Bennett, 1993 | ● | ○ | ● | ○ | ● | ● | ◌ | ● |
| Weber, 1991 | ● | ● | ● | ● | ● | ● | ◌ | ● |
| Lewis, 1983 | ● | ○ | ● | ○ | ● | ● | ◌ | ● |
| Spinapolice, 1982 | ● | ○ | ● | ○ | ● | ● | ◌ | ● |

Q1: Were patient’s demographic characteristics clearly described?; Q2: Was the patient’s history clearly described and presented as a timeline? Q3: Was the current clinical condition of the patient on presentation clearly described?; Q4: Were diagnostic tests or methods and the results clearly described?; Q5: Was the intervention(s) or treatment procedure(s) clearly described?; Q6: Was the post-intervention clinical condition clearly described?; Q7: Were adverse events (harms) or unanticipated events identified and described?; Q8: Does the case report provide takeaway lessons? ● = Yes; ○ = No; ᴓ = Unclear; ◌ = Not Available

**Table S6.** Risk of bias assessment for cohort studies (Methodological Index for Non-Randomized Studies (MINORS) tool).

| **Author**  **Year** | **Aim** | **Inclusion of consecutive patients** | **Prospective collection of data** | **Endpoints appropriate to the aim of the study** | **Unbiased assessment of the study endpoint** | **Follow-up period appropriate to the aim of the study** | **Loss to follow up less than 5%** | **Prospective calculation of the study size** | **Total** | **Risk of bias** |
| --- | --- | --- | --- | --- | --- | --- | --- | --- | --- | --- |
| Hantouli,  2023 | 1 | 1 | 0 | 2 | 2 | 2 | 2 | 0 | 10/16 | Low |
| Sultan,  2022 | 1 | 1 | 0 | 2 | 2 | 2 | 2 | 0 | 10/16 | Low |
| Arturo Diaz,  2020 | 2 | 2 | 1 | 2 | 2 | 2 | 2 | 0 | 13/16 | Low |
| Cheng,  2020 | 2 | 2 | 0 | 2 | 2 | 2 | 2 | 0 | 12/16 | Low |
| Barut,  2019 | 1 | 2 | 0 | 2 | 2 | 2 | 2 | 0 | 11/16 | Low |
| El Messidi,  2018 | 2 | 2 | 0 | 2 | 2 | 2 | 2 | 0 | 12/16 | Low |
| Sarici,  2018 | 2 | 2 | 0 | 2 | 2 | 2 | 2 | 0 | 12/16 | Low |
| Shigemi,  2018 | 1 | 2 | 0 | 2 | 2 | 2 | 2 | 0 | 11/16 | Low |
| Caliskan,  2017 | 2 | 1 | 0 | 2 | 2 | 1 | 2 | 0 | 10/16 | Low |
| Hedstrom,  2017 | 1 | 2 | 1 | 1 | 2 | 2 | 0 | 0 | 9/16 | Some concerns |
| Ilhan,  2016 | 1 | 2 | 0 | 2 | 2 | 2 | 2 | 0 | 11/16 | Low |
| Kolbeinsson,  2016 | 2 | 2 | 0 | 2 | 2 | 2 | 2 | 0 | 12/16 | Low |
| Alvarez Villasenor,  2007 | 1 | 2 | 1 | 2 | 2 | 2 | 2 | 0 | 12/16 | Low |
| Ibiebele,  2017 | 1 | 2 | 0 | 2 | 2 | 2 | 2 | 0 | 11/16 | Low |
| Cox,  2015 | 1 | 2 | 2 | 2 | 2 | 2 | 0 | 0 | 11/16 | Low |
| Jorge,  2015 | 1 | 2 | 0 | 2 | 2 | 2 | 0 | 0 | 11/16 | Low |
| Kocael,  2015 | 1 | 2 | 1 | 2 | 2 | 2 | 2 | 0 | 12/16 | Low |
| Paramanathan,2015 | 1 | 2 | 1 | 2 | 2 | 2 | 2 | 0 | 12/16 | Low |
| Weiner,  2015 | 1 | 2 | 0 | 2 | 2 | 2 | 2 | 0 | 11/16 | Low |
| Del Avila Silva,  2014 | 2 | 2 | 0 | 2 | 2 | 2 | 2 | 0 | 12/16 | Low |
| Ibitoye,  2014 | 2 | 1 | 1 | 2 | 2 | 0 | 2 | 0 | 10/16 | Low |
| Veerappan,  2013 | 1 | 2 | 0 | 2 | 2 | 2 | 0 | 0 | 9/16 | Some concerns |
| Othman,  2012 | 1 | 1 | 0 | 2 | 2 | 2 | 2 | 0 | 10/16 | Low |
| Dhupar,  2010 | 2 | 2 | 1 | 2 | 2 | 2 | 2 | 0 | 13/16 | Low |
| Acs,  2009 | 1 | 2 | 2 | 2 | 1 | 2 | 2 | 0 | 12/16 | Low |
| Chiappetta Porras, 2009 | 1 | 2 | 1 | 2 | 2 | 2 | 2 | 0 | 12/16 | Low |
| Merabishvili,  2007 | 1 | 2 | 1 | 2 | 2 | 2 | 2 | 0 | 11/16 | Low |
| Lu,  2004 | 1 | 2 | 0 | 2 | 2 | 2 | 2 | 0 | 11/16 | Low |
| Al Akeely, 2003 | 2 | 2 | 0 | 2 | 2 | 2 | 2 | 0 | 12/16 | Low |
| Chiedozi,  2001 | 1 | 2 | 0 | 2 | 2 | 2 | 2 | 0 | 11/16 | Low |
| Barone,  1999 | 1 | 2 | 2 | 2 | 2 | 2 | 2 | 0 | 13/16 | Low |
| Cosenza,  1999 | 1 | 2 | 0 | 2 | 2 | 2 | 2 | 0 | 11/16 | Low |
| Sungler,  1999 | 1 | 1 | 2 | 2 | 2 | 2 | 2 | 0 | 12/16 | Low |
| Glascow,  1998 | 2 | 2 | 0 | 2 | 2 | 2 | 2 | 0 | 12/16 |  |
| Ramirez,  1998 | 1 | 2 | 0 | 2 | 2 | 2 | 2 | 0 | 11/16 | Low |
| Elamin Ali,  1997 | 2 | 1 | 0 | 2 | 2 | 2 | 2 | 0 | 11/16 | Low |
| El Masry,  1997 | 2 | 0 | 0 | 1 | 2 | 0 | 2 | 0 | 7/16 | Some concerns |
| Reyes,  1997 | 0 | 2 | 0 | 2 | 2 | 2 | 2 | 0 | 10/16 | Low |
| Abuabara, 1996 | 1 | 1 | 0 | 2 | 2 | 2 | 2 | 0 | 10/16 | Low |
| Davis,  1995 | 1 | 2 | 0 | 2 | 2 | 2 | 2 | 0 | 11/16 | Low |
| Swisher,  1994 | 1 | 2 | 0 | 2 | 2 | 0 | 2 | 0 | 9/16 | Some concerns |
| Eleding,  1993 | 2 | 0 | 0 | 2 | 2 | 2 | 2 | 0 | 10/16 | Low |
| Dixon,  1987 | 1 | 1 | 0 | 2 | 2 | 2 | 2 | 0 | 10/16 | Low |
| Landers,  1987 | 1 | 2 | 0 | 2 | 2 | 2 | 2 | 0 | 11/16 | Low |
| Hiatt,  1986 | 1 | 1 | 0 | 2 | 2 | 2 | 2 | 0 | 10/16 | Low |
| Greene,  1963 | 1 | 1 | 0 | 2 | 2 | 2 | 2 | 0 | 10/16 | Low |

Methods

The Methodological Index for Non-Randomised Studies (MINORS) was used for the risk of bias assessment of observational studies [ref]. MINORS is a valid instrument designed to assess the methodological quality of non-randomised studies, whether comparative or non-comparative. MINORS' domains are scored as 0 if they are not reported, 1 when they have been reported but with inadequate details, and 2 when they have been reported while providing adequate information. The global ideal score is 16 for noncomparative studies, MINORS <6 were considered as high risk of bias, while MINORS between 6-9 were considered as moderate risk of bias [ref].Results

Almost all studies were considered low risk of bias (Table S4), except one study was considered having a moderate risk of bias. The most frequent missing information was the prospective sample size calculation (0/5, 0%).

**Table S7.** Quality rating for the analyses of outcomes based on GRADE.

| **Outcome** | **Studies (participants)** | **Risk of bias** | **Inconsistency** | **Indirectness** | **Imprecision** | **Overall certainty** | **Operative** | **Non-operative** | **RR (95% CI)** |
| --- | --- | --- | --- | --- | --- | --- | --- | --- | --- |
| Caesarean section | 6 observational studies (8,561) | Serious | Very serious (I²=91%) | Not serious | Serious | ⨁◯◯◯ Very low | 132/2813 (4.7%) | 998/5748 (17.4%) | 0.49 (0.18–1.34) |
| Preterm delivery | 7 observational studies (6,799) | Serious | Serious (I²=51%) | Not serious | Serious | ⨁◯◯◯ Very low | 51/2564 (2.0%) | 93/4235 (2.2%) | 1.22 (0.60–2.46) |
| Abortion | 4 observational studies (6,565) | Serious | Not serious (I²=46%) | Not serious | Very serious | ⨁◯◯◯ Very low | 16/2477 (0.6%) | 30/4088 (0.7%) | 0.90 (0.26–3.18) |
| Postpartum haemorrhage | 2 observational studies (5,965) | Serious | Very serious (I²=97%) | Serious* | Very serious | ⨁◯◯◯ Very low | 59/2679 (2.2%) | 288/3286 (8.8%) | 0.41 (0.09–1.92) |
| Venous thromboembolism | 2 observational studies (18,225) | Serious | Not serious (I²=0%) | Serious† | Serious | ⨁⨁◯◯ Low | 38/9442 (0.4%) | 83/8783 (0.9%) | 0.55 (0.37–0.82) |
| Other complications | 8 observational studies (18,824) | Serious | Serious (I²=75%) | Serious‡ | Serious | ⨁◯◯◯ Very low | 516/9723 (5.3%) | 657/9101 (7.2%) | 0.96 (0.69–1.34) |

CI: Confidence interval, GRADE: Grading of Recommendations, Assessment, Development and Evaluations, RCT: randomised controlled trial, RR: Risk ratio, TAC: transabdominal cerclage, TVC: transvaginal cerclage

* Inconsistency: similarity of point estimates, extent of overlap of confidence intervals, and statistical criteria including tests of heterogeneity and *I*^2^

** Indirectness: differences in population, intervention and outcome and indirect comparisons

*** Imprecision: examination of 95% CIs and optimal information size

**†** For the aforementioned outcomes plausible residual confounding (e.g. inadequate follow-up period, demographic characteristics of the included populations including age, distribution of high risk HPV subtypes) would potentially suggest spurious effect.
